# Impact of cardiopulmonary bypass on acute kidney injury following coronary artery bypass grafting: a matched pair analysis

**DOI:** 10.1186/1749-8090-9-20

**Published:** 2014-01-18

**Authors:** Simon Schopka, Claudius Diez, Daniele Camboni, Bernhard Floerchinger, Christof Schmid, Michael Hilker

**Affiliations:** 1 Department of Cardiothoracic Surgery, University Medical Center Regensburg, Franz-Josef-Strauss-Allee 57, 93057 Regensburg, Germany

**Keywords:** Cardiopulmonary bypass, Coronary artery bypass grafting, Acute kidney injury

## Abstract

**Background:**

Postoperative Acute Kidney Injury (AKI) after coronary artery bypass grafting (CABG) is a common complication associated with significant morbidity and mortality. Cardiopulmonary bypass (CPB) is accepted to contribute to the occurrence of AKI and is of particular importance as it can be avoided by using the off-pump technique. However the renoprotective properties of off-pump (CABG) are controversial. This analysis evaluates the impact of cardiopulmonary bypass on renal function.

**Methods:**

A matched-pair analysis of 1428 patients undergoing coronary artery bypass grafting was conducted. The patients were stratified according to their preoperative renal function and to risk factors for postoperative AKI. The development of the glomerular filtration rate (GFR) from before surgery until hospital discharge was analyzed. Incidence of AKI were analyzed. Furthermore the impact of CPB duration on postoperative GFR was assessed.

**Results:**

The occurrence of AKI increases the risk of thirty-day mortality (odds ratio of 4.3). The postoperative GFR decreases significantly after coronary artery bypass grafting but does not differ between onpump and offpump CABG (60.2 ± 24.5 vs 60.7 ± 24.8; p = 0.54). No difference regarding the incidence (26.6% vs 25%) and severity of AKI between cardiopulmonary bypass and the off-pump technique could be found. Duration of cardiopulmonary bypass does not correlate with the decline in postoperative glomerular filtration rate (Pearson Product Moment Correlation; p > 0.050).

**Conclusion:**

Neither the mere use nor duration of cardiopulmonary bypass proofed to be a risk factor for developing postoperative AKI in CABG patients with a comparable preoperative risk profile for postoperative renal dysfunction. Furthermore, the severity of postoperative AKI is not affected by the use of cardiopulmonary bypass.

## Background

Postoperative acute kidney injury (AKI) is one of the most frequent and serious complications following coronary artery bypass grafting. Depending on the specific definition, acute kidney injury occurs in up to 30% of patients undergoing coronary artery bypass grafting [1]. Development of kidney injury is associated with high short-term and long-term mortality, a more complicated hospital course, and a higher risk for infectious complications [2]. Even minimal changes in serum creatinine that may occur in the postoperative period are associated with a substantial decrease in survival [3]. In addition to preoperatively existing renal dysfunction, peripheral artery disease and diabetes as well as age, the technique and duration of cardiopulmonary bypass are considered as risk factors for developing AKI. Off-pump coronary artery bypass (OPCAB) grafting eliminates the need for cardiopulmonary bypass and, as such, is assumed to reduce AKI. However, previous studies provided conflicting evidence to support this hypothesis [3-5].

The purpose of this study was to understand the impact of cardiopulmonary bypass on postoperative AKI, based on the current AKI definition. Therefore, incidence and severity of AKI were assessed by analyzing OPCAB versus conventional coronary bypass (CCB) grafting. To this end matched pairs were created according to preoperative renal function and risk factors for AKI.

## Methods

Institutional approval was obtained (Institiutional Review Board of University Medical Center of Regensburg), and the need for informed consent was waived. The surgeries took place in our institution between 2004 and 2010. Data were obtained from our institutional prospectively maintained database. Patients operated on using the OPCAB technique represent all OPCAB patients in our institution during this time period. All surgeries were performed by the one surgeon. Exclusion criteria for the OPCAB technique were emergency procedures accompanied by hemodynamic instability. Patients operated on using cardiopulmonary bypass were assigned to the OPCAB patients according to the matching criteria described in the following paragraph. These patients were selected from all CABG patients in our institution during the aforementioned time period and were operated on by several surgeons.

### Matching of patients

Cases were selected from 714 patients operated using the off-pump technique. The control group consisted of 714 patients operated using CPB. An individual 1:1 matching was conducted using the following variables:

Preoperative stage of glomerular filtration rate as defined by the Kidney Disease Outcome Quality Initiative.

Preoperative ejection fraction (EF) grouped into normal EF (>50%), moderate impaired EF (30%-50%), severe impaired EF (<30%).

Diabetes, age, gender.

Patients with preoperative dialysis were excluded from the analysis.

Structural equality of cases and controls was analysed using fisher exact test for nominal data and the paired t test for continous data.

Tightness of fit concerning the preoperative glomerular filtration rate referred to the stages defined by the Kidney Disease Outcome Quality Initiative, controls were matched to cases in the same stage. Stages are defined as stage 1 (> 90 ml/min/m^2^), stage 2 (30–89 ml/min/m^2^), stage 3 (15–29 ml/min/m^2^) and stage 4 (< 15 ml/min/m^2^). Tightness of fit concerning the preoperative ejection fraction referred to the stages normal EF (>50%), moderate impaired EF (30%-50%), severe impaired EF (<30%). Matching concerning the age was done by the same year of life.

### Anesthesia and surgical techniques

Standardized anesthetic protocol included a low dose of narcotics, inhalation drugs and paralytic agents. Intraoperative hemodynamic monitoring consisted of permanent arterial blood pressure, central venous pressure and an electrocardiogram. During OPCAB procedures systolic arterial pressure was kept above 100 mmHg. Periods of controlled systolic pressure below 100 mmHg, when preparing the aorta for proximal anastomosis, were kept as short as possible. During CCB procedures a non-pulsatile roller pump established a blood flow of 2.4 ml/min/m^2^. The mean arterial pressure was kept above 60 mmHg. All patients were operated on via median sternotomy. In most OPCAB procedures the left anterior descending of the patient was revascularized first, followed by revascularization of the lateral and the inferior wall. Exposing the lateral and inferior walls of the heart while maintaining stable hemodynamics was supported by means of a deep stitch and a sling. Coronary shunts were inserted routinely whenever possible. Proximal anastomosis was completed in a disease-free aortic segment using the Heartstring device as previously described in detail [6]. In CCB procedures a two-stage cannula was used for venous drainage from the right atrium, whereas a 22F aortic cannula was employed for the distal ascending aorta. Following the cross-clamping procedure one shot of crystalloid HKT Bretschneider cardioplegia or of Calafiore blood cardioplegia was administered to initiate cardiac arrest. In cases of minimal extracorporal circulation (MECC) the priming volume was 500 ml, consisting of 20% Mannitol and Perfuflac solution (B. Braun, Melsungen, Germany). All MECC patients received antegrade multidose blood cardioplegia to induce cardioplegic arrest [7].

### Definition of AKI and indication for renal replacement therapy (RRT)

Postoperative AKI and its stages were diagnosed in line with the glomerular filtration rate (GFR) criteria of the current Acute Kidney Injury Network definitions, AKI was diagnosed by an increase of serum creatinine of greater than or equal to 50% compared to preoperative values. Severity of AKI was classified as stage 1 (serum creatinine increase by 50 to 100%), stage 2 (serum creatinine increase by 101 to 200%) and stage 3 (serum creatinine increase by more than 200% or the need for renal replacement therapy, RRT) [8]. RRT in the course of AKI was initiated for the following indications: pulmonary edema, oliguria defined as urine output of less than 0.5 ml per kg of body weight, metabolic acidosis, hyperkalemia and uremia not responding to conventional treatment.

### Calculation of glomerular filtration rate

The glomerular filtration rate was estimated according to the simplified, recalculated equation derived from the Modification of Diet in Renal Disease study (MDRD): eGFR [ml/min/1.73 m^2^] = 175 × (serum creatinine [mg/dl]) - 1.154 × (age [years]) - 0.203 × (0.742 in females) × (1.212 in African Americans) [9].

### Statistics

All statistical analysis were done with IBM SPSS Statistics 20.0 or STATA Data analysis and statistical software. Data are represented as the mean ± standard deviation (SD) unless otherwise indicated. After testing for normal distribution, continuous data were compared using the Student’s t-test for paired data or the Wilcoxon test for paired data. Potential risk factors were coded as present or absent and evaluated by univariate analysis. Univariate testing of categorical variables with binary outcome between two groups was performed with the McNemar’s test for paired data.

Univariate inter-group comparison of categorical variables with three ordered levels was done with Fleiss-Everitt test.

Correlation analysis of continous data were performed using the Pearson Product Moment Correlation. A p-value of less than 0.05 was considered significant.

## Results

After matching data from 714 OPCAB and 714 CCB patients were analyzed. There were no significant differences with regard to gender, age, prevalence of diabetes, congestive heart failure, additive Euroscore, chronic kidney disease, and preoperative renal function as measured by serum creatinine and the estimated glomerular filtration rate (Table 1). Significant differences were found on the number of coronary vessels affected and reoperations. In the CCB group more patients were suffering from three-vessel disease. Renewed surgeries were more frequent in the OPCAB group.

**Table 1:** Preoperative patient data

	**OPCAB**	**CCB**	**P**
Patients (n)	714	714	1.0
Female (n)	165	165	1.0
Age	67 ± 9.4	67 ± 9.6	0.6
eGFR > 90 ml/min/m^2^	201	201	1.0
GFR 60–89 ml/min/m^2^	345	345	1.0
GFR 30–59 ml/min/m^2^	146	146	1.0
GFR 15–29 ml/min	13	13	1.0
GFR <15 ml/min	9	9	1.0
EF <30%	31	31	1.0
EF 30-50%	285	285	1.0
EF >50%	398	398	1.0
Euroscore add*	3.4, (0.88-62.0)	3.6, (0.88-55.5)	0.6
Diabetes mellitus	234	234	1.0
One vessel disease	75	9	<0.001
Two vessel disease	158	79	<0.001
Three vessel disease	481	626	<0.001
Reoperation	39	17	<0.003
PAD	76	76	1.0

Univariate analysis of preoperative patient data of OPCAB vs CCB patients. Values are presented as mean ± SD; * Data presented as median, minimum, maximum. OPCAB, off- pump coronary artery bypass; CCB, on-pump coronary artery bypass; eGFR, estimated glomerular filtration rate (ml/min/1.73 m^2^); EF ejection fraction; PAD peripheral artery disease.

**Figure 1:**
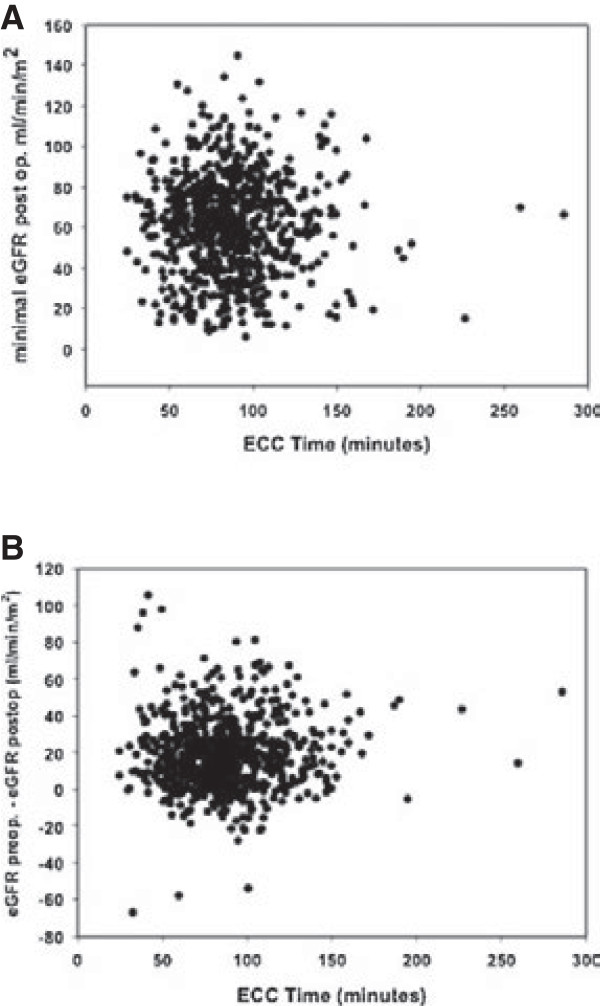
**Scattered plots of extracorporeal circulation duration versus minimal postoperative eGFR (A) and versus difference in preoperative and minimal postoperative eGFR (B) showing no correlation.** Pearson Product Moment Correlation > 0.050.

Postoperative results are shown in Table 2. In terms of thirty-day mortality there were no differences between the OPCAB and the CCB group. Occurrence of AKI significantly increased thirty-day mortality, adding up to 7.5%, whereas a thirty-day mortality of 1.7% was measured for patients without AKI (odds ratio of 4.3, 95% confidence interval, CI, of 2.39 to 8.02, p < 0.001). Regarding renal outcome the postoperative eGFR dropped significantly in both groups as shown in Figure 1. Renal function recovered in most patients (Figure 1), however, 235 (32.9%) patients in the OPCAB group and 191 (26.7%) in the CCB group were discharged from hospital with a worse GFR stage than at admission.

**Table 2:** General and renal postoperative outcome

	**OPCAB**	**CCB**	**p**	**Odds ratio**	**95% conf. int.**
Renal replacement therapy (n; %)	13 (1.8)	21 (2.9)	0.2295	1.62	0.77; 3.51
eGFR preoperatively ml/min/1.73 m^2^	81.5 ± 60.5	81.4 ± 62.7	0.093		-7.78; 0.46
Minimal eGFR postoperatively ml/min/1.73 m^2^	60.7 ± 24.5	60.2 ± 24.8	0.54		3.62; 7.7
eGFR at Discharge ml/min/1.73 m^2^	70.9 ± 26.8	72.4 ± 25.9	0.027		-32.91; -18.8
30d Mortality (n; %)	18 (2.5)	26 (3.6)	0.28	1.47	0.76; 2.90
Extracorporeal circulation (min.)		86.7 ± 29.4			
Aortic clamping Time (min.)		51.5 ± 18.1			
Duration of surgery (min.)	155 (55–384)	188 (84–633)	<0.001		-39.13; -29.2
Number of distal anastomosis	2.04 ± 0.78	3.04 ± 0.87	<0.001		-1.1; -0.91
Transfusion requirement (n; %)	323 (45.2)	378 (52.9)	0.0031	1.45	1.13 ;1.87
24 h chest tube loss (ml)	597 ± 599	759 ± 638	<0.001		-227.1; -91.2

AKI developed in 25% of OPCAB and 26.6% of CCB patients. Renal replacement therapy was necessary in 1.8% (n = 13) of OPCAB and 2.9% (n = 21) of CCB cases (p = 0.22). Analyzing the severity of AKI in both groups, again no significant difference was discovered (Table 3).

**Table 3:** Analysis of incidence of and stages of acute kidney injury

			
Acute Kidney Injury (n; %)	179 (25)	190 (26.6)	
Stage 1, n (%)	81 (11.3)	63 (8.8)	
Stage 2, n (%)	69 (9.6)	70 (9.8)	
Stage 3, n (%)	32 (4.4)	44 (6.1)	P = 0.5088

Cardiopulmonary bypass has been further analyzed as a risk factor for developing AKI in CCB group patients. Neither duration of cardiopulmonary bypass nor cross- clamp time showed any predictive value of these variables for developing AKI. A correlation analysis of bypass time and cross-clamping with minimal postoperative eGFR using the Pearson Product Moment Correlation and testing for inverse linear regression revealed no relation between duration of cardiopulmonary bypass and postoperative GFR.

## Discussion

The principal findings of this analysis are (1) in this patient cohort, use and duration of extracorporeal circulation did not influence postoperative renal function, (2) eGFR drops significantly following CABG, independent of the applied technique (OPCAB or CCB); (3) postoperative development of eGFR and incidence of acute kidney injury do not differ significantly between OPCAB and CCB CABG.

Matched pairs analysis provide the opportunity to rule out confounders by providing structural equality of cases and controls concerning known risk factors. This structural equality proved to be given in this analysis since there are neither numeral nor statistical differences in the preoperative risk factors for developing postoperative AKI.

The occurrence of acute kidney injury after cardiac surgical procedures significantly influences mortality and morbidity of patients. Mortality associated with the development of AKI is as high as 60% in some studies but likely averages 15 to 30%, depending on the definition of AKI and the postoperative period studied. In patients requiring dialysis, mortality is uniformly high in all studies and averages 60 to 70% [2,10,11]. A multivariate analysis, adjusted to comorbid factors, identified the occurrence of AKI as an independent determinant of the risk of death with an odds ratio of 7.9 [12]. This is consistent with the outcome of our analysis.

Postoperative deterioration of renal function not only affects early postoperative mortality but also worsens long-term results of CABG patients [1,3]. The importance of AKI is additionally emphasized by a high incidence of this complication. The incidence of AKI varies considerably in a range from 1 to 30%, depending on the underlying definition [1]. In this analysis, the current definition by the Acute Kidney Injury Network was used. Our results are in line with published data [13].

The marked deterioration in outcome and the high incidence of postoperative AKI are reasons to search for risk factors and intervention options. There is no comprehensive mechanism explaining renal failure associated with cardiac surgery, instead it is the result of a complex interplay of a number of related factors [14,15]. Clinical variables that are related to renal ischemia caused by arteriosclerosis and exacerbation by perioperative reduction of cardiac output, hypotension, and resultant hypoperfusion are accepted as significant perioperative risk factors. The majority of risk factors for the development of postoperative AKI is patient-related and, thus, no perioperative intervention options are available [14]. The typical risk factors in literature correspond to those observed in this analysis.

Use of extracorporeal circulation is believed to be associated with a deterioration of renal function [16,17]. This is of particular importance since applying the OPCAB technique makes this a preventable risk factor for AKI. Thus, OPCAB appears to be a logical step toward preventing postoperative AKI. However, the role of the OPCAB technique in the development of AKI is discussed controversially in literature [3-5], even large, controlled randomized studies comparing OPCAB with CCB CABG present varying results [ROOBY, Coronary], yet it is recommended for patients with impaired renal function in the current guidelines for myocardial revascularization [18]. Conflicting results in published studies in this respect may be due to the fact that there are numerous risk factors that predispose to the development of postoperative AKI. Since preexisting kidney injury is accepted as important risk factor for AKI stratifying patients with respect to preoperative renal function appears to be essential. As the most sensitive marker of renal impairment, the GFR is especially suitable for this purpose [9].

The present study ensures preoperative comparability, whereas most of the published studies did not stratify their patient cohort as per preoperative renal function, [3,19-22] or postoperative renal function was not primary or secondary endpoint of the study.

Not only the use of cardiopulmonary bypass, but also bypass time has been attributed to reduced postoperative renal function [23,24]. Accordingly, an inverse correlation between bypass time and postoperative GFR would be expected. This correlation, however, could not be found for bypass or clamping time. This further underlines the independence of AKI from cardiopulmonary bypass. Certainly these results are only valid for the range of CPB times investigated in this analysis. Duration of CPB and postoperative renal function are discussed controversially. Whereas Mancicni et al. published results comparable to those obtained in this analysis, a metanalysis of Kumar et al. identified duration of CPB as independent risk factor of AKI. Again stratification of preoperative renal function is an important difference between these analysis.

### Limitations

The present study is limited by its retrospective nature and the relevant restrictions. As a result no standardized treatment approach could be adopted for the study, therefore, bias of the results owing to differences in treatment strategy as well as experience and skill of surgeons and anesthesiologists cannot be excluded. Furthermore, the GFR was derived by the MDRD formula, which was not designed for acute renal impairment. Such inaccuracies in the calculation of the postoperative GFR in acute deterioration of renal function cannot be prevented. The study is further limited by the fact that the compared cohorts differ in the number of diseased coronary vessels. Further differences between case and control cohort are presented by transfusion requirement, which was indentified as independent risk factor for development of AKI. However patients undergoing CCB CABG required more transfusions than OPCAB patients, such this rather underlines the indpendence of CPB and AKI. Analysis of the impact of duration of extracorporeal circulation on postoperative GFR is limited by the fact that this analysis is performed on a population selected for a matched pairs analysis. A further possible limitation is the use of conventional extracorporeal circulation and MECC in the CCP group, however prior comparison of MECC and conventional ECC analysing the incidence of AKI and RRT could not find significant differences between the two types of extracorporeal circulation [17].

## Conclusions

In summary, this analysis revealed incidence and risk increase by postoperative AKI in a sufficiently large patient population. The collected data are consistent with published literature, with one exception relating to the use of extracorporeal circulation. Neither use nor duration of cardiopulmonary bypass were found to be independent risk factors for postoperative deterioration of the GFR and the occurrence of AKI. Subject to the limitations of the study the results allow the conclusion that the impact of cardiopulmonary bypass on postoperative renal function in coronary patients is negligible.

## Competing interests

The authors declare that they have no competing interests.

## Authors’ contributions

SS: study design, data analysis, writing of the manuscript. CD: study design, data analysis and correction of the manuscript. DC: data interpretation, helping drafting the manuscript. BF: data collection and interpretation, helping drafting the manuscript. CS: study design, data interpretation, correction of the manuscript. MH: study design, data collection, analysis and interpretation, correction of the manuscript. All authors read and approved the final manuscript.
